# Supplementary material for: A Hybrid Epithelial to Mesenchymal Transition in Ex Vivo Cutaneous Squamous Cell Carcinoma Tissues
Source: Int J Mol Sci. 2022 Aug 16;23(16):9183. doi: 10.3390/ijms23169183 (PMC9408944; doi:10.3390/ijms23169183)
Supplement: Supplementary file 1 [file ijms-23-09183-s001.zip › ijms-1805175-supplementary.pdf]

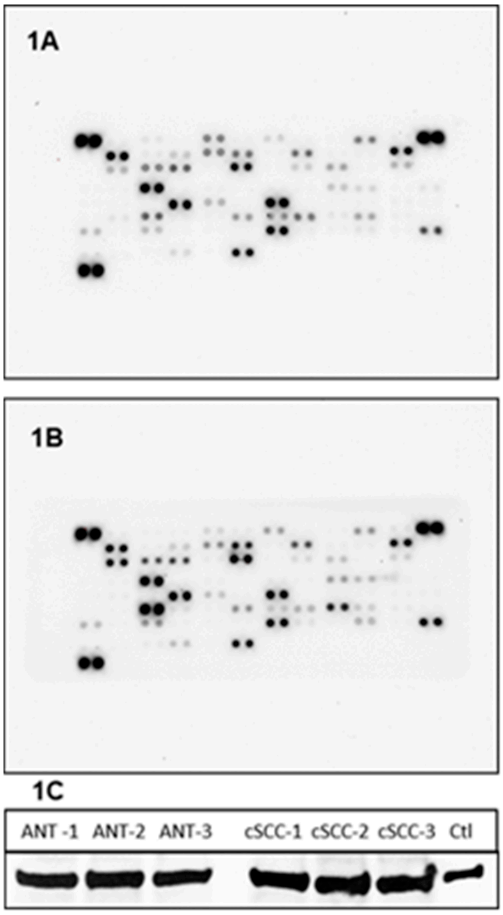

Figure S1. Representative dot blots showing expression of 84 oncogenic proteins from ANT and cSCC tissues.

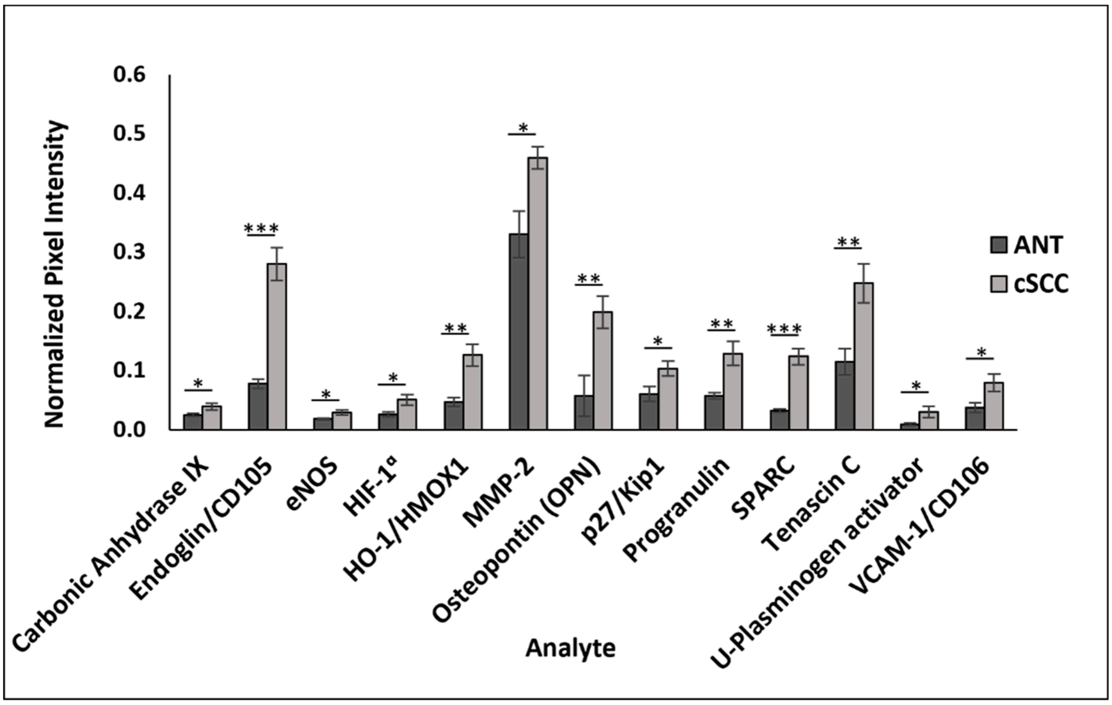

Figure S2. Protein expression using a Proteome Profiler Oncogenic Array.

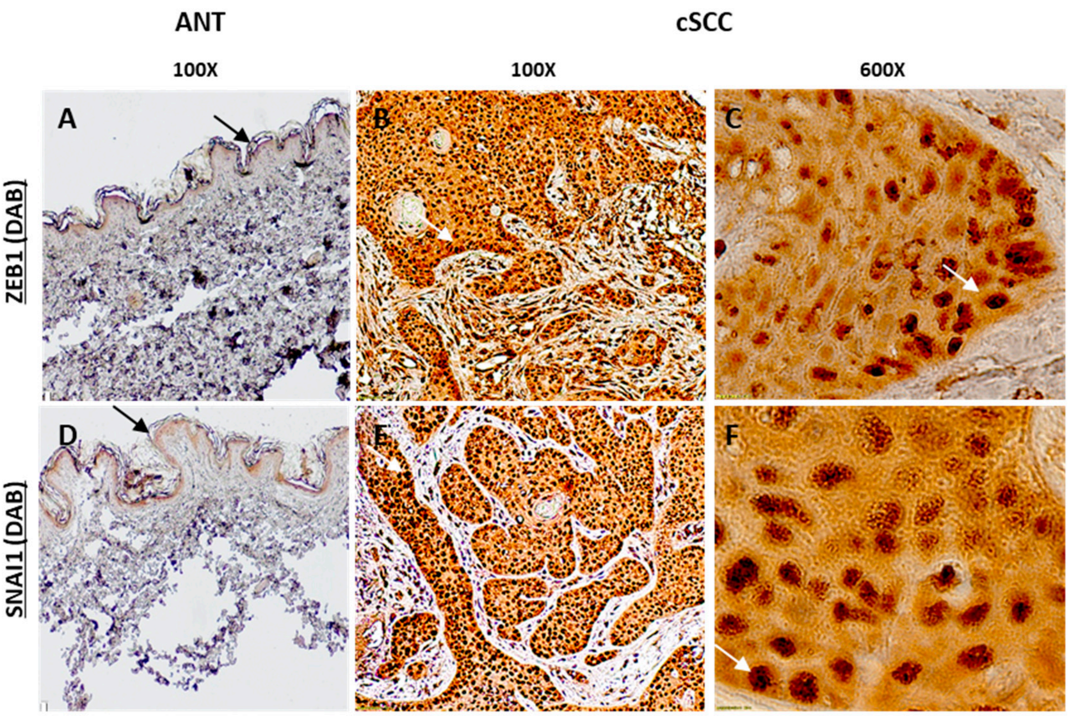

Figure S3. Immunohistochemistry of normal (ANT) and cancer (cSCC) tissues for ZEB1 and SNAI1.

Table S1. Demographics of patient-derived samples obtained from ALBR®.

Group	N	Diagnosis	Sex	Median Age	Location		
cSCC	74	SCCis (46); SCC (6); Invasive (22)	Male (43); Female (31)	69	Face, Scalp & Neck (51)	Trunk&Shoulder (10)	Extremities (13)
ANT	71	SCCis (44); SCC (5); Invasive (22)	Male (40); Female (31)	69	Face, Scalp & Neck (42)	Trunk&Shoulder (11)	Extremities (18)
